# Multifocal Abscesses, Necrotizing Fasciitis, Iron Deficiency Anemia, and Hypophosphatemia Induced by Ferric Carboxymaltose Infusions: Report of a Case of Hereditary Hemorrhagic Telangiectasia

**DOI:** 10.7759/cureus.44020

**Published:** 2023-08-24

**Authors:** Luana A Trevise, Melissa P Lopes Vieira Pinto, Gabriela Hasselmann, Bruna C Lammoglia, Thatiany P Leal, Nilton Salles Rosa Neto

**Affiliations:** 1 Rheumatology, Universidade Santo Amaro, São Paulo, BRA; 2 Center for Rare and Immune Disorders, Hospital Nove de Julho, São Paulo, BRA

**Keywords:** necrotizing fasciitis, abscess, intravenous iron replacement, ferric derisomaltose, ferric saccharate, ferric carboxymaltose, hereditary hemorrhagic telangiectasia, iron deficiency anemia

## Abstract

Hereditary hemorrhagic telangiectasia (HHT) is a rare autosomal dominant vascular dysplasia in which disrupted angiogenesis leads to increased formation of mucocutaneous telangiectasias or major vascular malformations. Iron deficiency anemia and recurrent abscesses are commonly reported in these patients, reinforcing screening and targeted therapies for these conditions. We report a 50-year-old man with HHT affected by repeated episodes of iron deficiency anemia secondary to recurrent epistaxis requiring frequent intravenous iron infusions. He eventually developed hypophosphatemia and hyperphosphaturia secondary to ferric carboxymaltose. He also had a history of recurrent multifocal abscesses, including a severe presentation of necrotizing fasciitis, requiring multiple surgical interventions. Despite the identification of hypogammaglobulinemia, only after consistent dental treatment and antibiotic prophylaxis did the abscesses stop recurring. We highlight the need for careful consideration of all possible complications inherent to the disease itself but also those related to comorbidities or existing treatments.

## Introduction

Hereditary hemorrhagic telangiectasia (HHT) is a rare autosomal dominant genetic disease in which pathogenic variants detected most frequently in the endoglin (ENG), activin type II receptor type 1 (ACVRL1), or SMAD4 genes disrupt angiogenesis, leading to increased formation of mucocutaneous telangiectasias, or major vascular malformations. Less frequently reported variants associated were found in GDF and RASA1 genes [1-3].

Patients with suspected HHT can be diagnosed by the Curaçao criteria [4], where the fulfillment of three or more criteria provides a definite diagnosis of HHT, whereas the presence of two criteria constitutes a possible diagnosis of HHT. The four items considered are a) a history of recurrent epistaxis, b) the presence of mucocutaneous telangiectasia, c) the presence of visceral lesions (pulmonary, gastrointestinal, hepatic, spinal, or cerebral arteriovenous malformations (AVM)), and a positive first-degree family history may point to the diagnosis [1-3].

The formation of abnormal and easily ruptured blood vessels most often leads to recurrent bleeding and eventually to iron deficiency and anemia. The average onset of epistaxis is around 12 years of age [2]. Patients may also be prone to developing abscesses, stroke, portal or pulmonary hypertension, or high-output heart failure [1,5].

Screening for iron deficiency is recommended in all adults with HHT, regardless of symptoms, due to an estimated prevalence of greater than 60% [6]. Iron replacement may vary according to the degree of deficiency, recurrence of bleeding, and patient preference [7].

Patients with HHT have a significantly increased risk of polymicrobial infections and abscesses, especially in the context of pulmonary AVM [6]. Studies report a 6-8% prevalence of brain abscesses in specialized centers, but the frequency of abscesses in other parts of the body has not been determined. Antibiotic prophylaxis before dental and surgical procedures is necessary for these patients to reduce the risk of infectious complications [6].

Intravenous iron-induced hypophosphatemia has been reported with all parenteral iron formulations. However, it seems not to be related to the iron molecule itself, but to the molecules complexed with it [8,9].

Herein, we report a patient diagnosed with HHT and two commonly described complications - recurrent abscesses and iron deficiency anemia - and discuss the peculiarities related to the clinical presentation and management options, including antimicrobial prophylaxis, and the choice of different options for intravenous iron supplementation for this patient care.

## Case presentation

This work was approved by the Universidade Santo Amaro Ethics Review Board, São Paulo, Brazil, under number 5.305.324 on March 22, 2022. The subject read and signed informed consent for publication. The procedures were followed in accordance with the ethical standards of the responsible committee on human experimentation and with the Helsinki Declaration of 1975, as revised in 1983.

**Figure 1:**
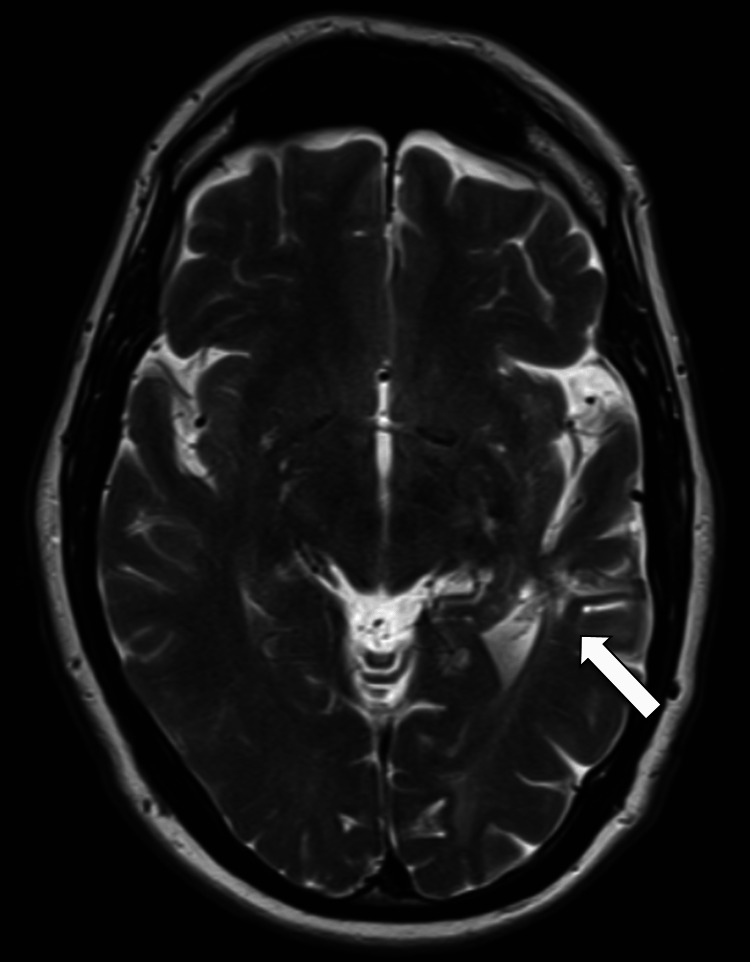
Magnetic resonance imaging of the head (T2W post-contrast) depicting the area of encephalomalacia of the left temporal lobe (arrow) – previous cerebral abscess.

A 50-year-old man was hospitalized in September 2015 with a 10-day history of fever associated with pain and swelling in his right hand. He was admitted under rheumatological care for suspicion of arthritis. He reported a family history of HHT and a personal history of recurrent epistaxis, generalized cutaneous and mucosal telangiectasias, pulmonary and one cerebral AVM, and a previous episode of brain abscess treated with antibiotics (Figure 1 shows the area of encephalomalacia, secondary to previous brain abscess). The patient's clinical picture is consistent with being affected by HHT. He also reported smoking and a history of nephrolithiasis. Genetic evaluation was not performed.

**Figure 2:**
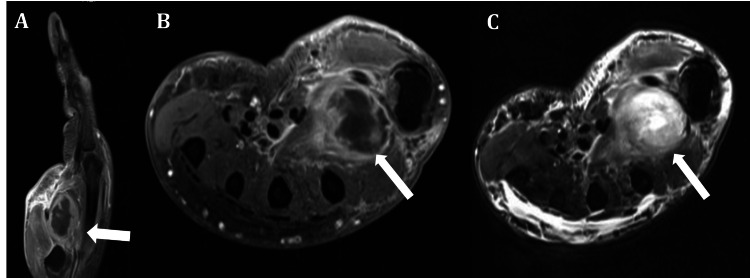
Magnetic resonance imaging. A. T1W post-contrast sagittal view, B. T1W post-contrast transverse view, and C. T2W post-contrast transverse view, depicting abscess at the thenar eminence of the right hand (arrows).

Imaging revealed a 4.5 mL collection of intramuscular fluid on the dorsal aspect of the hand suggestive of an abscess without evidence of osteomyelitis (Figure 2). He was started on ceftriaxone and daptomycin and underwent surgical drainage. No microorganisms were identified, but he recovered well after completing the antibiotic course.

**Figure 3:**
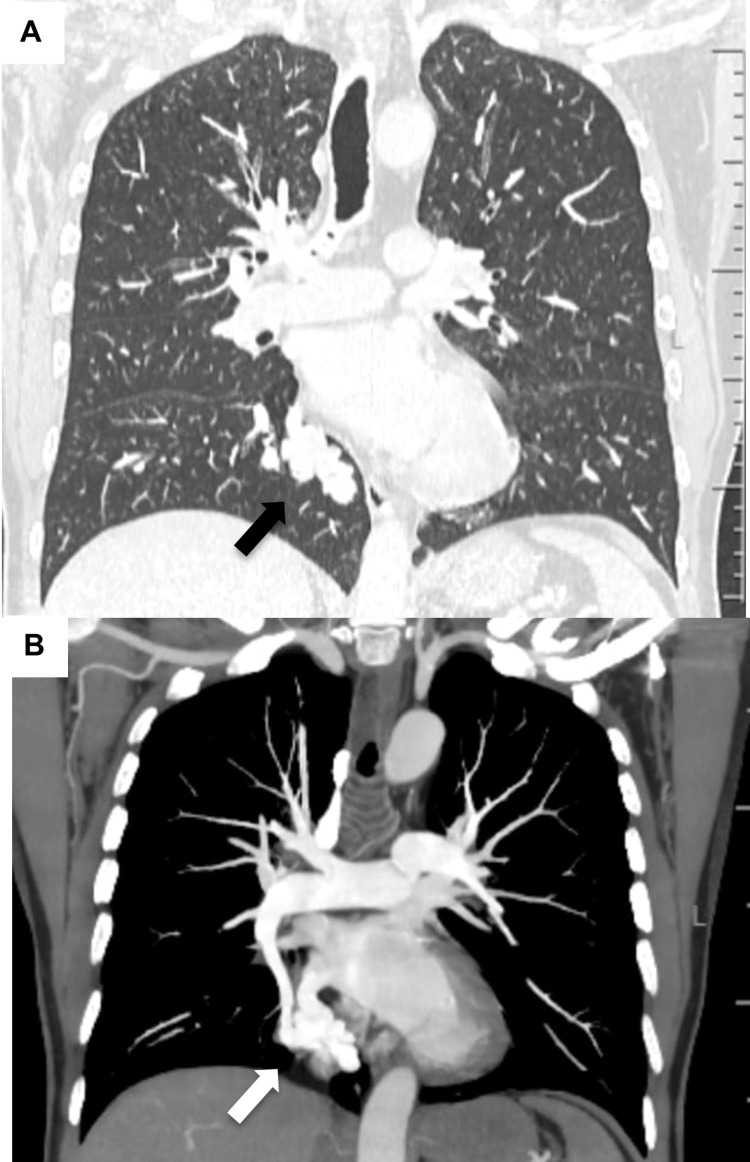
Computed tomography angiography of the chest (A. lung window, B. mediastinal window) depicting the arteriovenous malformation in the right lower lobe (arrows).

Additional imaging was performed and revealed AVM in the brain (right subcortical temporal lobe) - image not available - and lung. Computed tomography angiogram disclosed an AVM in the distal medial projection in the right lower lobe, measuring 3.6 cm x 4.3 cm between the medial basal pulmonary arterial branch (caliber of about 0.6 cm) and the posteroinferior pulmonary vein on that side (with 1.2 cm) (Figure 3).

During hospitalization, he reported chest pain and was transferred to the intensive care unit because of non-ST-segment elevation myocardial infarction. His coronary angiogram was normal, but a magnetic resonance imaging (MRI) of his heart revealed the affected area (image not available). His hemoglobin at the time was 10.3 g/dL (range 13-14.9). He was discharged without aspirin after careful consideration of the 2013 publication from Devlin et al. [10] and in view of the patient's high risk of bleeding.

**Figure 4:**
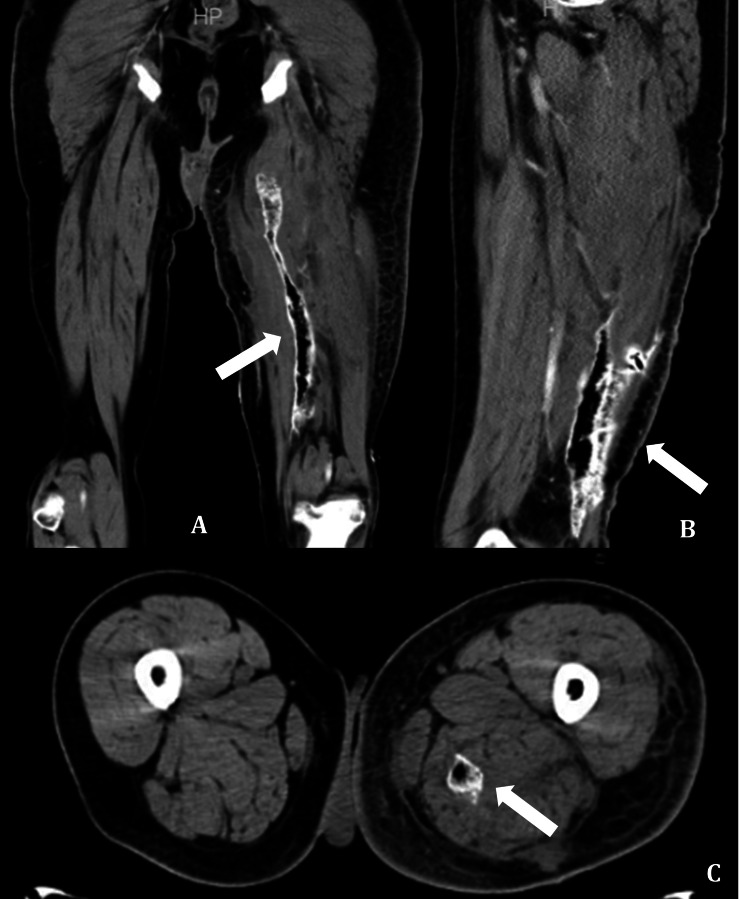
Magnetic resonance imaging of the thighs (T2W post-contrast), depicting an extensive area of necrotizing fasciitis on the posteromedial aspect of the left thigh (arrow). A. coronal view, B. sagittal view, and C. transverse view.

One month later (October 2015), he developed severe pain and swelling in his left thigh. Imaging revealed a 7.0 x 2.6 x 2.4 cm collection of fluid near the left adductor magnus (Figure 4). Ceftriaxone and daptomycin were reinstituted, and a new surgical procedure was performed to drain the collection. This time, two different bacteria grew in the culture: Acinetobacter lwoffii and Klebsiella pneumoniae. Antibiotics were changed to polymyxin B, but he could not tolerate it because of severe paresthesia. Based on sensitivity, meropenem was selected. Despite the treatment, the infection progressed, and he developed necrotizing fasciitis. Linezolid and amikacin were added, and he underwent extensive surgical debridement.

**Figure 5:**
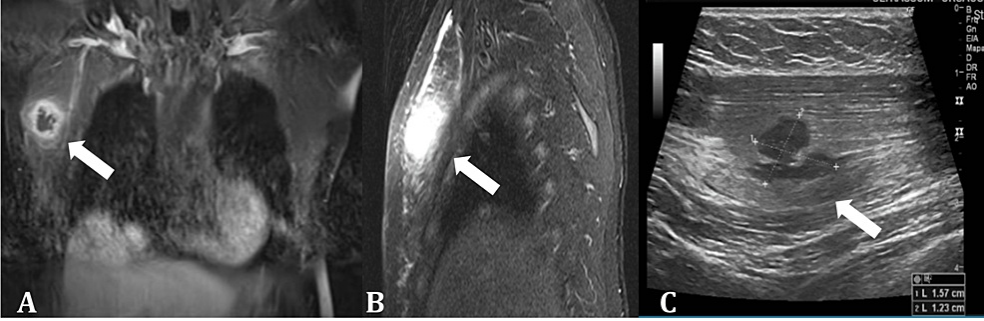
Magnetic resonance imaging of the thorax. A. T1W post-contrast coronal view, B. T2W post-contrast sagittal view, and C. Ultrasound gray scale, depicting the abscess at the right pectoralis major muscle (arrow).

At hospital discharge, he was maintained on oral ciprofloxacin to complete a prolonged period of treatment. During hospitalization, he was evaluated by a dentist, who identified severe periodontitis, and we proposed frequent dental cleaning, alongside amoxicillin prophylaxis and smoking cessation to prevent new bacteremia episodes and abscesses. He adhered to the proposed scheme, but in December 2016, he was admitted with an abscess in the right pectoralis major muscle that required surgical drainage (Figure 5). Again, no microorganisms were identified. Cefepime and daptomycin were used, and he recovered well. In none of the episodes was infective endocarditis diagnosed.

Of note, intermittent hypogammaglobulinemia was noted. Table 1 shows the serial measurements of immunoglobulin (Ig) levels. Due to the cessation of abscesses recurrence, the lack of evidence of other kinds of infections, and the IgG levels mostly above 250 mg/dL, human intravenous immunoglobulin was not recommended.

**Table 1:** Sequential serum immunoglobulin measurements (2016-2021).

	Reference range	2016	2017	2019	2021
IgG, mg/dL	700-1600	871	575	309	523
IgM, mg/dL	40-230	94	57	38.5	50.3
IgA, mg/dL	70-400	201	156	84.4	133.1

After this series of infections, he maintained the process of dental cleaning and prophylactic use of amoxicillin 2 g on the day of the procedure. This protocol has been used ever since, and no further infectious events were identified. Furthermore, endovascular treatment of the pulmonary AVM was not considered feasible at the moment, and there were no signs of hypoxemia or high-output cardiac failure. It is noteworthy that he continues to smoke despite several efforts to stop.

However, episodes of epistaxis remained a persistent problem with iron deficiency anemia, requiring recurrent intravenous infusions of iron, as oral supplementation was ineffective in maintaining normal hemoglobin and ferritin levels. The patient refused a trial of bevacizumab or thalidomide. Surgical procedures were performed on the nasal mucosa, but eventually bleeding would recur.

**Figure 6:**
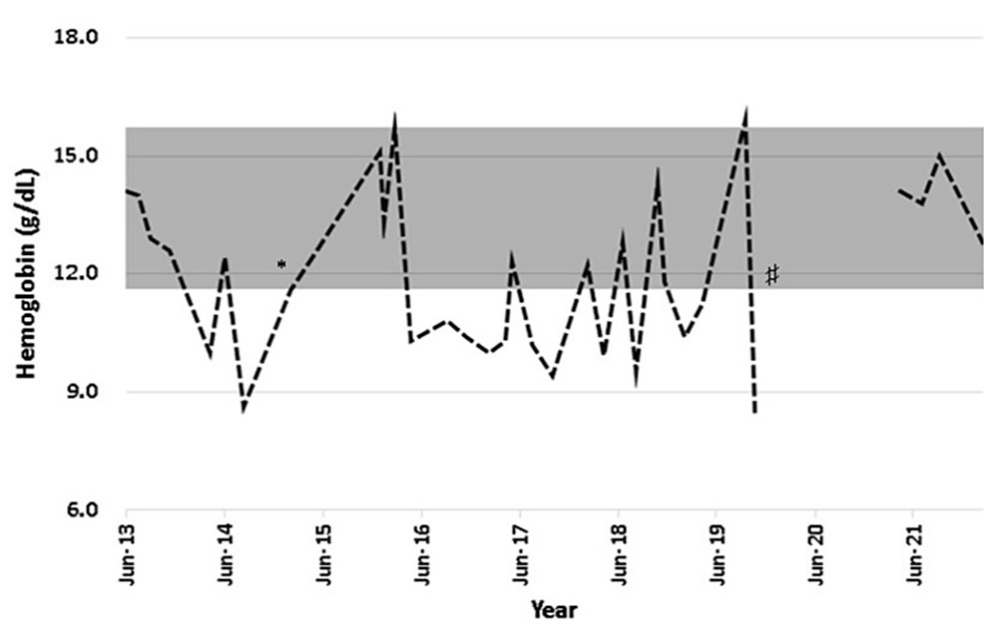
Sequential outpatient hemoglobin measurements, from 2013 to 2021, reference range 13.5-17 g/dL (shaded area); interval refers to the COVID-19 pandemic period. * ferric carboxymaltose treatment initiation; ♯ COVID-19 pandemic period.

**Figure 7:**
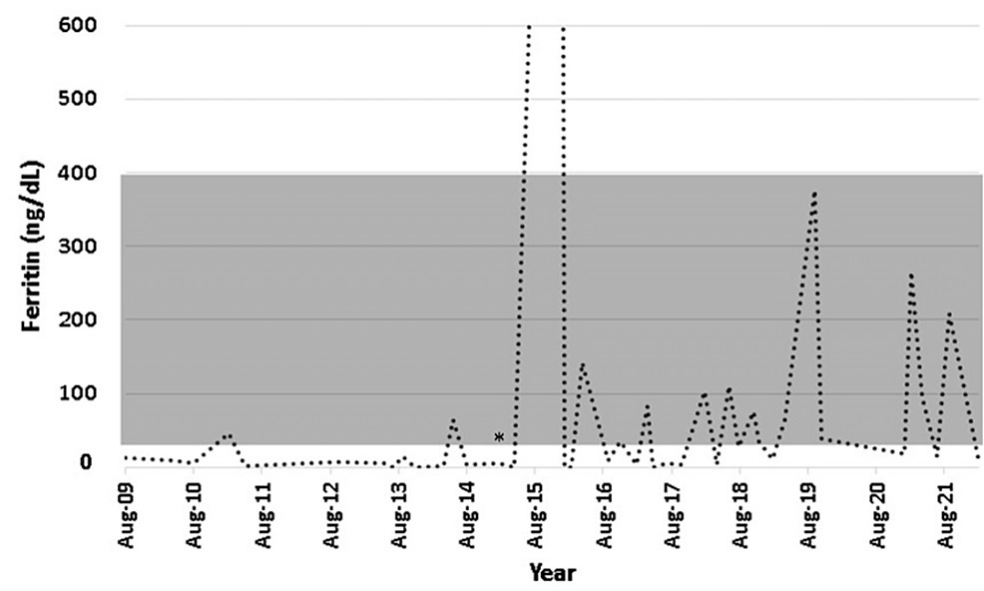
Sequential outpatient ferritin measurements, 2009 to 2021, reference range 30-400 ng/mL (shaded area). * ferric carboxymaltose treatment initiation.

He was treated with monthly infusions of 200 mg of ferric saccharate before the series of recurrent abscesses. Regrettably, the yield was low, and he required frequent infusions. During the 2015 hospitalization, he was treated with iron carboxymaltose 1,000 mg per month, and after discharge, he received a new infusion every time his transferrin saturation was below 15% or he had severe nasal bleeding. This approach permitted a reduction in the number of infusions. He also used tranexamic acid during the bleeding episodes, which would reduce blood loss for minor bleeds, but not for severe bleeds. Figures 6-7 show the sequence of outpatient hemoglobin (from 2013 to 2021) and ferritin (from 2009 to 2021) measurements, illustrating the recurrent iron deficiency anemia compatible with the clinical diagnosis of HHT.

Lately, chronic supplementation of ferric carboxymaltose (FCM) has been associated with fibroblast growth factor (FGF)-23-mediated hypophosphatemia and osteomalacia [9,11]. The patient was evaluated because of six years of regular infusions. He denied symptoms of myalgia, weakness, and bone pain and had normal alkaline phosphatase 90 U/L (range 40-150) and normal calcium 9.1 mg/ dl (range 8.8-10.4), but low phosphorus 2.1 mg/dL (range 2.3-4.3), low TmP/GFR (ratio of tubular maximum reabsorption of phosphate to the glomerular filtration rate) 2.29 mg/dL (range 2.60-3.80), low PTH 7.5 pg/mL (range 14-72), low 25-hydroxyvitamin D 19.3 ng/dL (range 30-60), and inappropriately normal FGF-23 84 RU/mL (range 26-110). There was no evidence of osteomalacia and a history of fractures, and he had normal bone density as assessed by dual X-ray absorptiometry (lumbar spine T-score of -0.7; right femoral neck T-score of -1.0; right hip total T-score of -0.4).

However, due to the persistent hypophosphatemia, the potential for developing osteomalacia, and the frequent need for iron replacement, the patient was transitioned to intravenous ferric derisomaltose (FDI). As of this moment, the yield is good, and there is no evidence of hypophosphatemia, hyperphosphaturia, or hyperphosphatasemia consistent with osteomalacia. The good results imply maintaining his hemoglobin levels above 12 g/dL, without evidence of dyspnea, lightheadedness, tachycardia, postural hypotension, or any other sign of clinically overt anemia. Considering that he keeps bleeding periodically, whenever his hemoglobin levels fall below 12 g/dL, he is scheduled to receive a dose of FDI. After this approach, he was hospitalized only once due to massive epistaxis and required blood transfusion and bilateral internal maxillary artery embolization. After approximately three months, epistaxis recurred.

## Discussion

This is a report on an adult patient with HHT and the particularities of his treatment regarding management decisions to reverse iron deficiency anemia and prevent further episodes of deep tissue abscesses.

Management of recurrent abscesses in HHT

Infections are commonly associated with HHT. A cohort of 357 patients presented 13.6% of infectious events, mostly extra-cerebral and largely related to Staphylococcus aureus [12]. Abscesses in HHT are mainly reported in the brain. Expert guidelines provide information on diagnosing, preventing, and treating this situation. However, little is published regarding the occurrence of abscesses in other parts of the body [12].

Regarding cerebral abscesses, the risk is estimated to be approximately 1,000 times greater for HHT patients than for the general population. Of note, among HHT type 1 patients with pulmonary AVM, the risk is estimated to be 400 times greater [12,13].

A recent Danish study of the prevalence of cerebral abscesses in the general population identified only two HHT patients in a cohort of eighty subjects. For this general population, antibiotic therapy was directed by the sensitivity pattern of the causative agent. Metronidazole, penicillin, and ceftriaxone were the most prescribed antibiotics, with an average length of treatment between four and 12 weeks. Treatment consisted of a combination of intravenous and oral preparations. Interestingly, adjuvant steroid treatment was given in 63.8% of cases in general [13].

For the prevention of cerebral abscesses, antibiotic prophylaxis is recommended prior to dental procedures [3]. Investigation and treatment of pulmonary AVM is recommended [7,14,15]. Interventional radiology is the preferred approach when the afferent artery is at least 1-3 mm according to the expertise of the radiologist. However, there is a greater chance of recanalization, and a close follow-up is warranted. In the event of refractory situations or extensive lesions, a surgical approach or even lung transplantation can be considered [2].

Our patient had evidence of pulmonary AVM and a history of cerebral abscess, consistent with the common presentation in subjects with HHT. However, he later developed multiple deep soft tissue abscesses and eventually had an episode of necrotizing fasciitis, but recovered after prolonged hospitalization. Regular dental cleaning and antibiotic prophylaxis prevented the recurrence of the abscesses in our patient.

Correction of the pulmonary AVM was discussed, but due to previous experience with intervention in a similar lung lesion in an affected sibling with posterior recanalization, the treatment was not performed.

Immunological disturbances in HHT

Guilhem et al. [16] identified abnormalities of adaptive immunity in patients with HHT. Their subjects exhibited low numbers of CD4+ and CD8+ T-cells and natural killer (NK) cells. The authors also reported increased levels of IgG and IgA, but low levels of IgM.

Conversely, our patient had intermittent low levels of IgG that could not be attributed to drugs or infections usually associated with reduced levels. His IgA levels remained within normal limits, and IgM levels were mostly within the reference range, except for one measurement in 2019. This immune abnormality was not present at the most critical moment of his illness, but a close follow-up revealed IgG levels below the lower limit of normal for several years after the episode of necrotizing fasciitis. Fortunately, the institution of broad-spectrum antibiotic therapy and rapid surgical debridement managed to reverse the dramatic situation.

After regular dental treatment and antibiotic prophylaxis, the abscesses ceased, and chronic parenteral immunoglobulin replacement was unnecessary. However, we cannot exclude the supposition that intermittently low immunoglobulin levels could have contributed to the severity of our patient's infection, but specific cellular immunodeficiency was not evaluated.

Management of iron deficiency anemia in HHT

Iron deficiency anemia is a common complication in HHT. The etiology of anemia, in most cases, is chronic epistaxis and, less commonly, gastrointestinal bleeding. Concomitant shortened intravascular red blood cell survival consistent with microangiopathic hemolysis may contribute to a subset of patients with anemia disproportionate to bleeding. Classic signs and symptoms include fatigue, reduced exercise tolerance, tachycardia, restless legs syndrome, and hair loss [17].

It is recommended that all adult patients, regardless of symptoms, and all children patients with recurrent bleeding and/or symptoms of anemia due to HHT be evaluated for iron deficiency anemia. Testing comprises complete blood count and ferritin and transferrin saturation [6].

Oral iron replacement is recommended as an initial therapeutic consideration. Guidelines underline that intravenous iron may be considered the first-line option in patients with severe anemia or in those in whom oral replacement is ineffective. Additional recommendations are referral to hematology and red blood cell transfusion when needed [3].

Guidelines recommend initiating oral iron replacement with 35-65 mg of elemental iron daily. If inadequate, increasing the daily dose may be considered. If not tolerated, alternative oral iron preparations could be tried. During the follow-up, it is important to monitor the effectiveness of the response, aiming at transferrin saturation >20% and ferritin >50 ng/mL and an increase of >1.0 g/dL in hemoglobin levels. Patients should be reassessed periodically (6-8 weeks) [3].

If hemoglobin does not rise to expected levels with oral iron replacement, or if there is contraindication to the oral route, severe anemia, low adherence, or adverse events, initiation of intravenous iron is recommended. Local guidelines and consultation with a hematologist should direct the amount and timing of supplementation. Regularly scheduled iron infusions may be necessary [18]. There are a number of standard iron preparations available for use, though the availability of each does vary between countries. Hemoglobin targets always need to be individualized in HHT, depending on the patient's symptoms, the severity of ongoing HHT-related bleeding, prior response to other therapies and oral iron supplements, the presence of comorbid conditions, and the acuity of the care environment. In some patients, supplementation with erythroid-stimulating agents (e.g., epoetin alfa, darbepoetin alfa) may be beneficial [17].

When bleeding is severe enough that intravenous iron or red blood cell transfusions are insufficient, consideration of antifibrinolytics such as tranexamic acid is an option [17]. Tranexamic acid was recommended to our patient, and it was able to reduce the amount of blood loss for minor bleedings, but not for major ones.

At the same time, surgical approaches are recommended to treat chronic bleeding such as epistaxis. These indications and treatments should be decided on the basis of clinical scores, most frequently when epistaxis is moderate or severe [3]. Treatment options are usually ablative therapies and include laser treatment, radiofrequency ablation, and sclerotherapy [3]. The patient underwent radiofrequency ablation, sclerotherapy, and, more recently, angiographic embolization. All procedures were effective only temporarily.

Iron-induced hypophosphatemia in HHT

Some intravenous iron formulations have been recognized to induce FGF23-mediated renal phosphate wasting syndrome. FCM and FDI are intravenous iron preparations that rapidly correct iron deficiency but can decrease serum phosphate concentrations by increasing renal phosphate excretion. The advantage of choosing these preparations is the reduced number of infusions needed when compared to ferric saccharate to reach target levels [8,9,18].

The incidence of hypophosphatemia is significantly higher after treatment with FCM than with FDI. Hypophosphatemia may persist beyond five weeks after initial administration of the drug. A comparative study of the incidence of hypophosphatemia after dosing of FCM and FDI revealed an 8% incidence of low phosphorus levels after FDI and 74% after FCM. Of note, only FCM was associated with phosphorus levels ≤1 mg/dL, in 11% of patients [8,9,11]. Thus, chronic exposure to FCM may lead more frequently to disturbances in bone and mineral metabolism than FDI. Physicians should be aware of this possible adverse event and prompt regular monitoring to prevention.

As reported in this case, our patient had recurrent epistaxis and was frequently iron-depleted. The need for frequent dosing of iron infusions, the detection of FGF-23-induced hyperphosphaturia and hypophosphatemia, and the potential risk of developing osteomalacia indicated that FDI was considered a better option in his case but without negligible risks.

## Conclusions

This case report illustrates a patient with HHT with repeated iron deficiency anemia secondary to recurrent epistaxis and multiple efforts to prevent blood loss. Recent publications highlighted the risk of hypophosphatemia and osteomalacia with chronic use of FCM and, according to his detailed clinical picture and patient preference, directed the therapeutic change to FDI. Of note, his metabolic panel normalized. In addition, the patient had pulmonary AVM and a history of multifocal abscesses, requiring several surgical procedures and the use of broad-spectrum antibiotics. A more severe presentation with necrotizing fasciitis eventually guided the identification of low levels of immunoglobulin G. It is not known if hypogammaglobulinemia could have played a role in the development of this severe infection. Regular scheduled dental cleaning and prophylactic use of antibiotics were sufficient to reduce the risk of recurrent abscesses, and there was no need for routine immunoglobulin replacement. The immunoglobulin profile in this patient is not consistent with previously published work and should encourage researchers to better understand this topic.
